# Applying Plant Hydraulic Physiology Methods to Investigate Desiccation During Prolonged Cold Storage of Horticultural Trees

**DOI:** 10.3389/fpls.2022.818769

**Published:** 2022-02-24

**Authors:** Rebecca A. Sheridan, Lloyd L. Nackley

**Affiliations:** ^1^Weyerhaeuser, Federal Way, WA, United States; ^2^North Willamette Research and Extension Center, Oregon State University, Corvallis, OR, United States; ^3^Department of Horticulture, Oregon State University, Corvallis, OR, United States

**Keywords:** arboriculture, chilling hours, water relations, non-structural carbohydrates, nursery management, urban forestry, vulnerability curves

## Abstract

Plant nursery production systems are a multi-billion-dollar, international, and horticultural industry that depends on storing and shipping live plants. The storage environment represents potentially desiccating and even fatal conditions for dormant, bareroot, and deciduous horticulture crops, like orchard trees, forestry trees, ornamental trees, and grapevines. When tree mortality is considered within a plant hydraulic framework, plants experiencing water stress are thought to ultimately die from hydraulic failure or carbon starvation. We hypothesized that the hydraulic framework can be applied to stored crops to determine if hydraulic failure or carbon starvation could be attributed to mortality. We used deciduous trees as model species because they are important horticultural crops and provide a diversity of hydraulic strategies. We selected cultivars from six genera: *Acer*, *Amelanchier*, *Gleditsia*, *Gymnocladus*, *Malus*, and *Quercus*. For each cultivar, we measured stem hydraulic conductance and vulnerability to embolism. On a weekly basis for 14 weeks (March–June), we removed trees of each cultivar from cold storage (1–2°C). Each week and for each cultivar, we measured stem water potential and water content (*n* = 7) and planted trees to track survival and growth (*n* = 10). At three times during this period, we also measured non-structural carbohydrates. Our results showed that for four cultivars (*Acer*, *Amelanchier*, *Malus, and Quercus*), the stem water potentials measured in trees removed from storage did not exceed stem *P*_50_, the water potential at which 50% of stem hydraulic conductivity is lost. This suggests that the water transport system remains intact during storage. For two cultivars (*Gleditsia* and *Gymnocladus*), the water potential measured on trees out of storage exceeded stem *P*_50_, yet planted trees from all weeks survived and grew. In the 14 weeks, there were no significant changes or directional trends in stem water potential, water content, or NSC for most cultivars, with a few exceptions. Overall, the results show that the trees did not experience detrimental water relations or carbon starvation thresholds. Our results suggest that many young deciduous trees are resilient to conditions caused by prolonged dormancy and validate the current storage methods. This experiment provides an example of how a mechanistically based understanding of physiological responses can inform cold storage regimes in nursery tree production.

## Introduction

The plant nursery industry produces billions of plants and trees, which are then shipped to global destinations (Haase et al., 2020). Making them unique among crops, plants grown in nursery production systems are often moved great distances in the middle of their lifecycle, which generates novel physiological stress to these otherwise stationary lifeforms. For this to be feasible, nursery professionals take advantage of natural phenophases, for example, harvesting and transferring dormant deciduous species. Many plant nurseries (hereafter referred to as nurseries) are located where favorable conditions and consistent seasonal cues induce winter dormancy. A wide variety of orchard species, forestry species, and ornamental species are produced in a nursery production system known as “bareroot” in which trees are excavated (aka “lifted”) in late fall and winter, stored at or just below freezing temperatures during winter, and shipped in the spring. Plants grown and sold in nursery systems must be able to withstand prolonged storage conditions without significant detrimental effects on growth and survival after planting (Maclennan and Fennessy, 2006; Dumroese et al., 2016). Decades of commercial trial and error have created rough guidelines for safe storage practices for bareroot plants. However, there is a need for relevant, mechanistic information about how long trees can tolerate cold storage without damage or death. We believe that investigating the impacts of desiccation and carbon depletion in cold storage through a plant hydraulic framework will help provide meaningful guidelines to this multi-billion-dollar annual industry (United State Department of Agriculture, 2020).

How long seedlings can be kept in cold storage is one of the key physiological questions for optimizing nursery production (Grossnickle et al., 2020). For example, more time in storage allows for the accumulation of chilling hours, which can lead to faster bud break and increased root growth after planting (Harris et al., 1993; Nanninga et al., 2017). The two key risks of storage are desiccation and carbon starvation due to respiration, in the absence of external causes of mortality, such as pests and pathogens (Mckay, 1997). Winter desiccation can impact performance in the growing season if the hydraulic function cannot be restored (Niu et al., 2017). Dormant trees rely on carbohydrate reserves for respiration and tissue development (Tixier et al., 2019). The physiological effects of cold storage have been well studied for conifer seedlings but are not understood as well for deciduous trees (Ritchie, 1982; Cannell et al., 1990; McKay, 1994). Developing parameters such as the length of time deciduous trees will tolerate cold storage conditions without a loss of plant vigor requires careful evaluation of multiple physiological traits (Jacobs et al., 2008). Even then, recommendations may vary by species and by variety or ecotype within species (Overton et al., 2013). Decisions about planting windows to avoid frost damage and ensure access to the planting site are made all the more difficult due to the unpredictable impacts of climate change (Chmura et al., 2011; Fargione et al., 2021). Nursery professionals may need to keep trees in cold storage for longer than is the current practice to respond dynamically to a changing climate.

A framework of tree mortality that considers the trade-offs between hydraulic failure and carbon starvation has been considered extensively (Sevanto et al., 2014; Adams et al., 2017). This “hydraulic framework” has been applied to explain tree mortality as a result of drought (Choat et al., 2018). Plant water relations and xylem function are typically studied in actively growing (i.e., non-dormant) seedlings and trees (Landsberg and Waring, 2017). However, the same physiological processes are important for the survival of dormant deciduous trees: the combined effects of water stress and low carbohydrate reserves can compromise winter cold tolerance, leading to a greater likelihood of mortality (Galvez et al., 2011). Water relations and carbon balance and the interactions of these fluxes coming out of the winter are also important to ensure function in the next growing season (Améglio et al., 2004; Tomasella et al., 2017).

Nursery production systems that use cold storage provide a unique opportunity to study the confounding physiological challenges that trees face through winter. Given the prevalence of cold storage in nursery production, it is important to understand the plant responses to develop research-based management practices that maintain plant health. Therefore, our objectives were to examine the patterns of plant water relations and NSC dynamics during storage for cultivated varieties (cultivars) of six genera of deciduous trees, *Acer, Gleditsia, Gymnocladus, Malus,* and *Quercus*. We selected these six genera as model species because they are high value and widely sold horticultural crops and provide a diversity of hydraulic strategies. We wanted to identify if these trees reach physiological thresholds that correlate with mortality during extended cold storage. If so, would the impacts from cold storage be evident in post-planting performance?

We had the following expectations for the plant physiological metrics that we measured in this experiment: stem water potential and stem water content would decline over an extended time in cold storage due to desiccation. Total stem NSC would also decline over time in cold storage, due to respiration. As a result of the compound impacts of desiccation and carbon depletion due to extended time in cold storage, height growth after outplanting would be diminished for trees that were in storage longer. In trees where the stem water potential dropped low enough to cause significant hydraulic failure in the xylem, we expected growth and survival after planting would be negatively affected.

## Materials and Methods

Starting March 19, 2020, we obtained trees from the cold storage facility at a commercial shade tree nursery in Canby, Oregon (45.21, −122.73). Retrieving trees from the storage facility continued every week until June 18, 2020, for a total of 14 weeks. The stored trees had been harvested in fall 2019 and washed of all soil in a process called bare-rooting. While in cold storage, the trees were stored at 1-2^o^ C. The coolers were turned off the week of June 8, 2020. Each week, we transported the trees to Oregon State University’s North Willamette Research and Extension Center (NWREC) in Aurora, Oregon (45.28, −122.75) for sampling and planting. Upon arrival at NWREC, we randomly sorted the trees into planting and destructive sampling cohorts. Ten trees were planted each week. For each of the seven trees selected for destructive sampling, we measured stem water potential, water content for the terminal stem segment, and non-structural carbohydrates in the terminal stem segment.

We evaluated six cultivars: Red Sunset^®^ maple (*Acer rubrum* “Franksred”); Prairie fire crabapple (*Malus* “Prairifire”); red oak (*Quercus rubra*); Skyline^®^ honeylocust (*Gleditsia triacanthos* “Skycole”); Autumn Brilliance^®^ serviceberry (*Amelanchier x grandiflora*); and Kentucky coffeetree (*Gymnocladus dioicus*). Due to logistical challenges, not all cultivars were planted at all weeks; additional details and planting and sampling timing can be found in Table 1.

**Table 1:** Cultivated species (cultivars) represented within this experiment and the corresponding dates for planting and NSC sampling.

Species	Cultivar name	Stock type	First planting date	Mid-season NSC sample date	Last planting date	Xylem anatomy
*Acer rubrum* ‘Franksred’	Red Sunset^®^ maple	4-foot whips; 4- and 5-foot branched saplings	March 19, 2020	April 30, 2020	June 18, 2020	Diffuse porous
*Amelanchier x. grandiflora*	Autumn Brilliance^®^ serviceberry	3- and 4-foot whips	April 2, 2020	April 30, 2020	May 21, 2020	Diffuse porous
*Gleditsia triacanthos* ‘Skycole’	Skyline^®^ honeylocust	3- and 4-foot whips	March 19, 2020	April 30, 2020	May 21, 2020	Ring porous
*Gymnocladus dioicus*	Kentucky coffeetree	4-foot whips	April 2, 2020	April 30, 2020	May 28, 2020	Ring porous
*Malus* ‘Prairifire’	Prairie fire crabapple	5-foot whips on hardy rootstock	March 19, 2020	April 30, 2020	May 21, 2020	Diffuse porous
*Quercus rubra*	Red oak	4-foot whips	March 19, 2020	April 30, 2020	June 18, 2020	Ring porous

Planting dates vary due to logistical issues that differed by cultivar. Xylem anatomy is also listed (InsideWood, 2004; Wheeler, 2011).

### Planting and Field Measurements

Each week, we planted 10 trees in a field at NWREC. The field has loam soil (47% sand; 32% silt; and 21% clay). Depending on the exact size of the weekly shipment, three to seven of the trees planted had the terminal shoot removed for other measurements described below; the remaining trees were planted without any pruning. We measured height at planting, and again on November 30, 2020, and calculated the incremental growth. Additionally, we monitored mortality and phenological development after planting.

### Environmental Conditions

**Figure 1:**
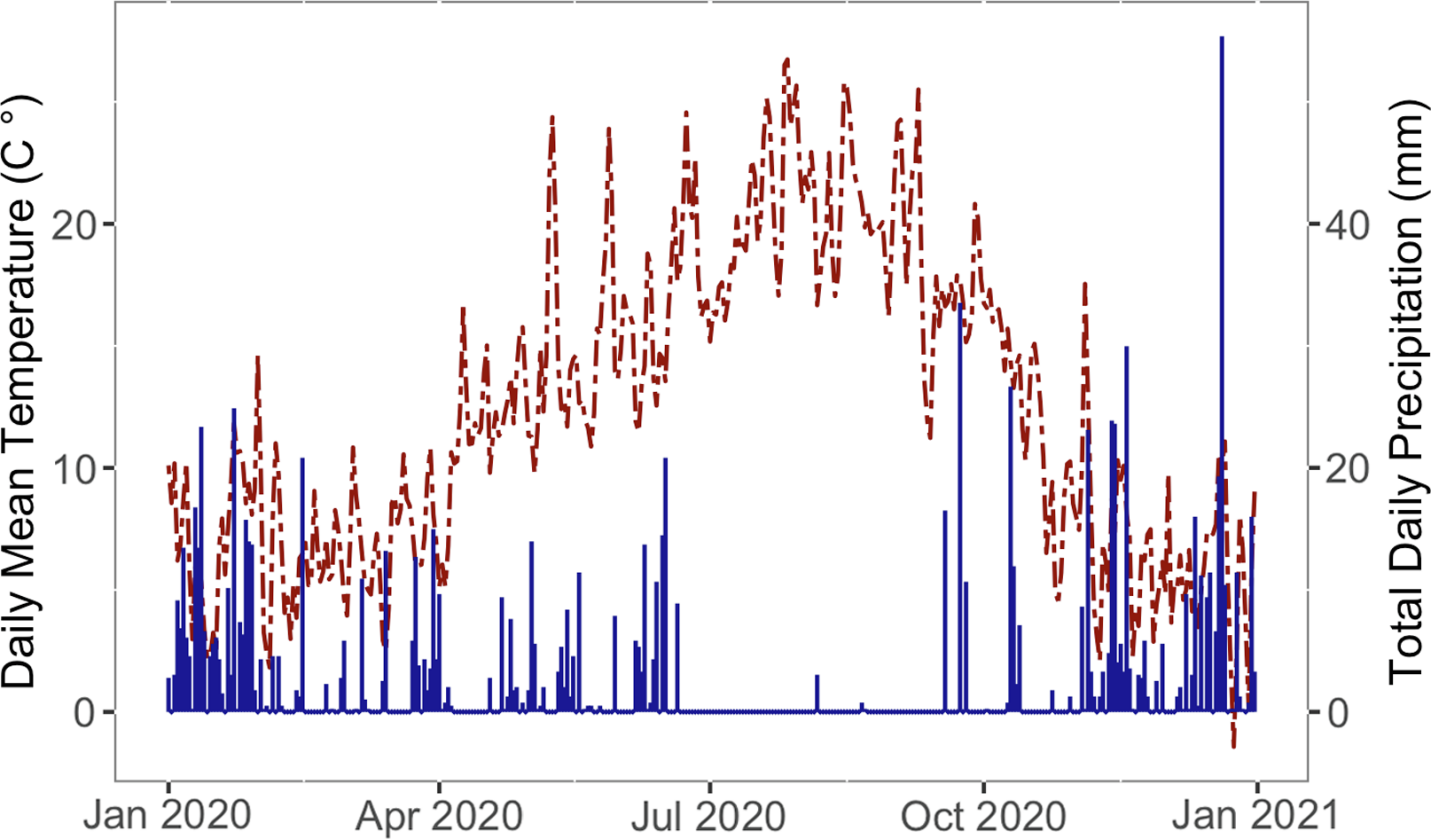
Daily mean temperature (left axis, red dashed line) and total daily precipitation (right axis, blue solid line) at the planting location over the first growing season after planting.

Temperature and precipitation were tracked by an U.S. Bureau of Reclamation AgriMet weather station that is located adjacent to the field and displayed in Figure 1 (AgriMet, 2016). Drip irrigation was installed within several weeks of each planting to mitigate any impacts of drought. By June 30, 2020, all trees were on drip irrigation.

### Physiological Measurements

Physiological measurements were made on seven individuals of each cultivar at each transplanting date.

#### Stem Water Potential

We measured midday stem water potential on the terminal shoot using a pressure chamber (Model 1505D-EXP, PMS Instruments, Albany, OR). Stem water potential can be reliably measured on dormant deciduous trees and used to track winter water stress (Milliron et al., 2018).

#### Water Content

We calculated the water content of the plant tissue using the following equation:


$$WC\;\left(\frac{g}{g}\right)=\frac{Fresh\;Weight-Dry\;Weight}{Dry\;Weight}$$

For stem water content, we weighed the same terminal stem segment used in the water potential measurements. Immediately after the stem water potential measurement, we put the stem segment into a plastic bag until measuring the fresh weight. We then dried the plant tissues in a drying oven at 75°C for 7 days, then weighed the samples.

#### Non-structural Carbohydrates:

Samples of the same stem segments used for water relations measurements were also used to analyze non-structural carbohydrates (NSC) by a lab at Oregon State University that specializes in this type of analysis. To extract the NSC, the samples were ground to a fine powder in a Wiley mill. The soluble carbohydrates were extracted in deionized water; starch was extracted with ethanol and an A-AMG (amyloglucosidase) enzyme solution. NSC content of the samples was quantified by measuring absorbance at 630 nm using a microplate multiscan reader (Leyva et al., 2008).

#### Stem Hydraulic Conductance and Vulnerability Curves

We measured stem hydraulic conductance and vulnerability to embolism on three samples for each of the species, except *Quercus*. We cut a stem segment of approximately 20 cm, less than 15 mm in diameter (small enough to fit the cavitation chamber), and without side branches from the middle of the tree. The initial cuts were made underwater because the unplanted trees could be laid within a basin of water. We immediately brought the stem segments into the lab, removed the bark, and recut the ends underwater. We then vacuum infiltrated stems overnight in 0.2 μm filtered deionized water to remove initial emboli.

#### Stem-Specific Conductivity

We measured stem conductance using the gravity-feed method with 0.2 μm filtered deionized water. We measured flow through the stem using an electronic balance (Explorer Ex224, Ohaus Corp., Parsippany, NJ) connected to a computer running the R-based program conductR (Smith, 2018). When the flow rate stabilized, we recorded flow as the average of the last four readings. To calculate hydraulic conductivity, we used the slope of the volume flow rate (kg s^−1^) vs. the pressure gradient (MPa m^−1^); then, to standardize to stem-specific conductivity, the hydraulic conductivity was divided by stem cross-sectional area (*k_s_*, kg m^−1^ s^−1^ MPa^−1^).

#### Vulnerability to Embolism

We used the air-injection method to measure stem vulnerability to embolism (Sperry and Saliendra, 1994). After the maximum stem conductivity was measured as described above, we inserted the stem segment into the cavitation chamber accessory for the PMS pressure chamber and pressurized for 10 min. After there were no longer bubbles exiting the stem due to pressurization, flow through the stem was measured again. The pressure was increased by 0.25 MPa intervals for *Gleditsia* and *Gymnocladus* and 0.5 MPa intervals for the other four cultivars. In between each pressure interval, stem-specific conductivity was again measured. Using conductR, the percent loss of stem-specific conductivity relative to maximum was plotted against the pressure applied by the pressure chamber to create vulnerability. We increased pressures in the cavitation chamber until 90% of stem-specific conductivity was lost, so the maximum pressurization varied for each cultivar. Vulnerability curves and water potential thresholds at which 12, 50, and 88% of stem-specific conductivity were lost (*P*_12_, *P*_50_, *P*_88_) were modeled using the R package fitPLC (Duursma and Choat, 2017).

#### Statistical Analysis

Each cultivar was analyzed separately for several reasons: the trees came from different nursery sources, the range of planting dates varied by species, and in a few cases, the sample size varied by species at planting. To test if there was a linear relationship between the week of planting and the plant physiological metrics, we used linear regression. The four physiological metrics, which were tested individually, were as: stem water potential, stem water content, total stem NSC, and height growth after planting. We used the software R version (4.0.2) for all statistical analyses.

## Results

The year 2020 started with warmer than average temperatures (Figure 1). By March 19th, when planting began, temperature closely followed the 30 year average [U.S.A. National Phenology Network (NPN), 2020]. In this region, most rainfall occurs from October through June, with a dry period from July to September. The region in which this project is located experienced moderate to severe drought conditions during the experiment (U.S. Drought Monitor, 2021). The trees were irrigated through the summer dry period.

**Figure 2:**
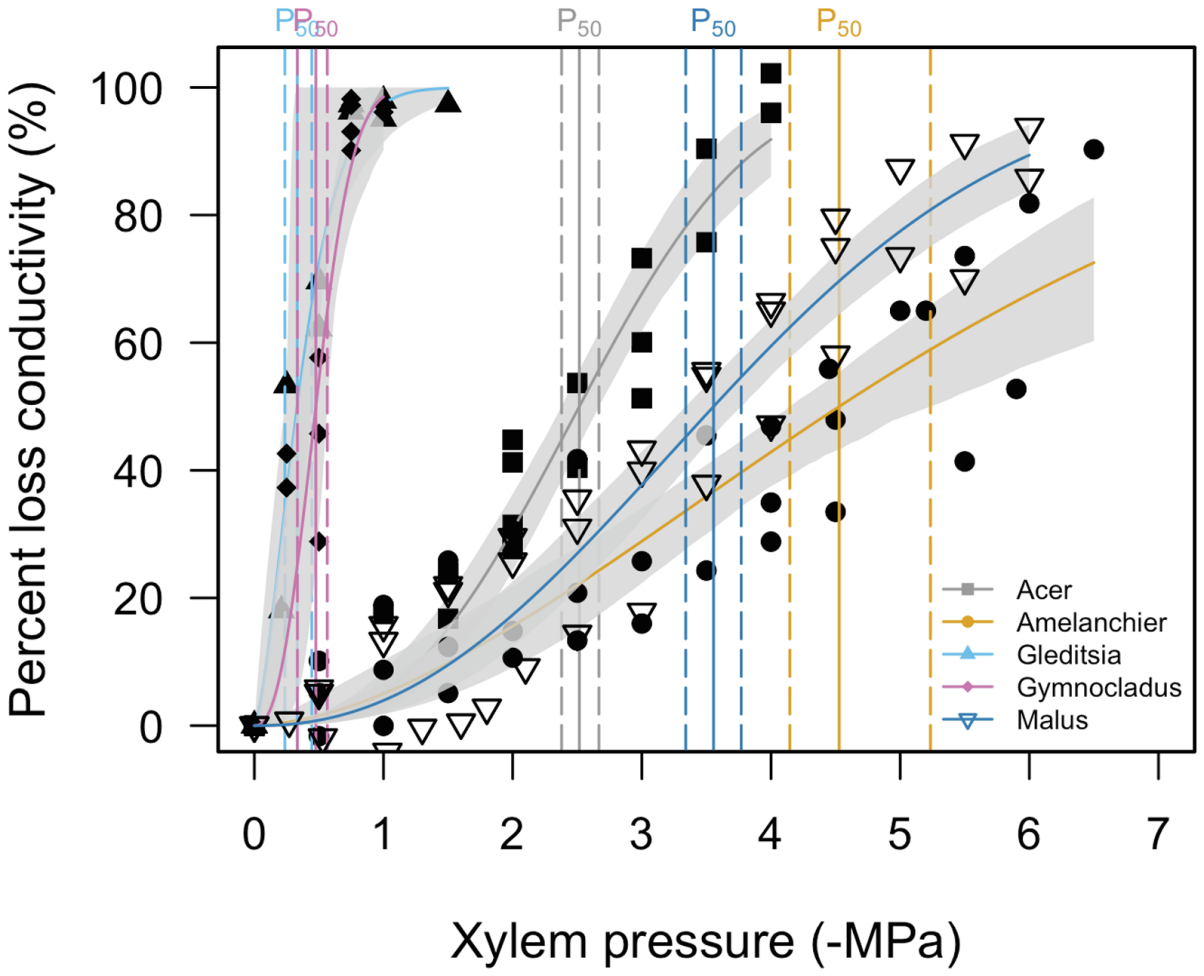
Stem vulnerability curves for five cultivars. Gray shading around each curve shows the 95% confidence interval of the curve (*n* = 3). Stem *P*_50_ for each species is indicated by a solid vertical line and 95% confidence intervals are indicated by a dashed vertical line. *Quercus* is not included (see text for details).

Stem *P*_12_, *P*_50_, and *P*_88_, as estimated from the vulnerability curves are reported in Table 2. Stem vulnerability curves showed two types of responses (Figure 2). *Gleditsia* and *Gymnocladus* demonstrated vulnerability to embolism at relatively mild water potentials (>−2.0 MPa). *Acer*, *Amelanchier*, and *Malus* were not as susceptible to embolism until more extreme water potentials (<−2.0 MPa). We did not build hydraulic vulnerability curves for the *Quercus* cultivar in this experiment due to the methodological challenges associated with hydraulic conductivity measurements with *Quercus* stems (Cavender-Bares and Holbrook, 2001). For this analysis, we use previously reported stem *P*_50_ values for *Q. rubra* (*P*_50_ = −2.5 MPa; Cochard and Tyree, 1990).

**Table 2:** Vulnerability curve parameters for five cultivars. Stem *P*_12_, *P*_50_, and *P*_88_ are modeled from the vulnerability curves shown in Figure 2.

	Stem *P*_x_	Estimate	95% Confidence Interval	
*Acer*	*P*_12_	−1.37	−1.12	−1.61
	*P*_50_	−2.52	−2.34	−2.69
	*P*_88_	−3.77	−3.42	−4.24
*Amelanchier*	*P*_12_	−1.70	−1.21	−2.21
	*P*_50_	−4.53	−4.16	−4.97
	*P*_88_	−8.66	−7.24	−11.38
*Gleditsia*	*P*_12_			
	*P*_50_	−0.33	−0.26	−0.40
	*P*_88_	−0.70	−0.57	−0.88
*Gymnocladus*	*P*_12_			
	*P*_50_	−0.60	NA	NA
	*P*_88_	−0.75	NA	NA
*Malus*	*P*_12_	−1.67	−1.40	−1.95
	*P*_50_	−3.56	−3.36	−3.76
	*P*_88_	−5.85	−5.35	−6.51

*N* = 3 for each cultivar. For Gleditsia and Gymnocladus, the stem P_50_ was higher than − 1 MPa and P_12_ was not estimated from the collected data.

**Figure 3:**
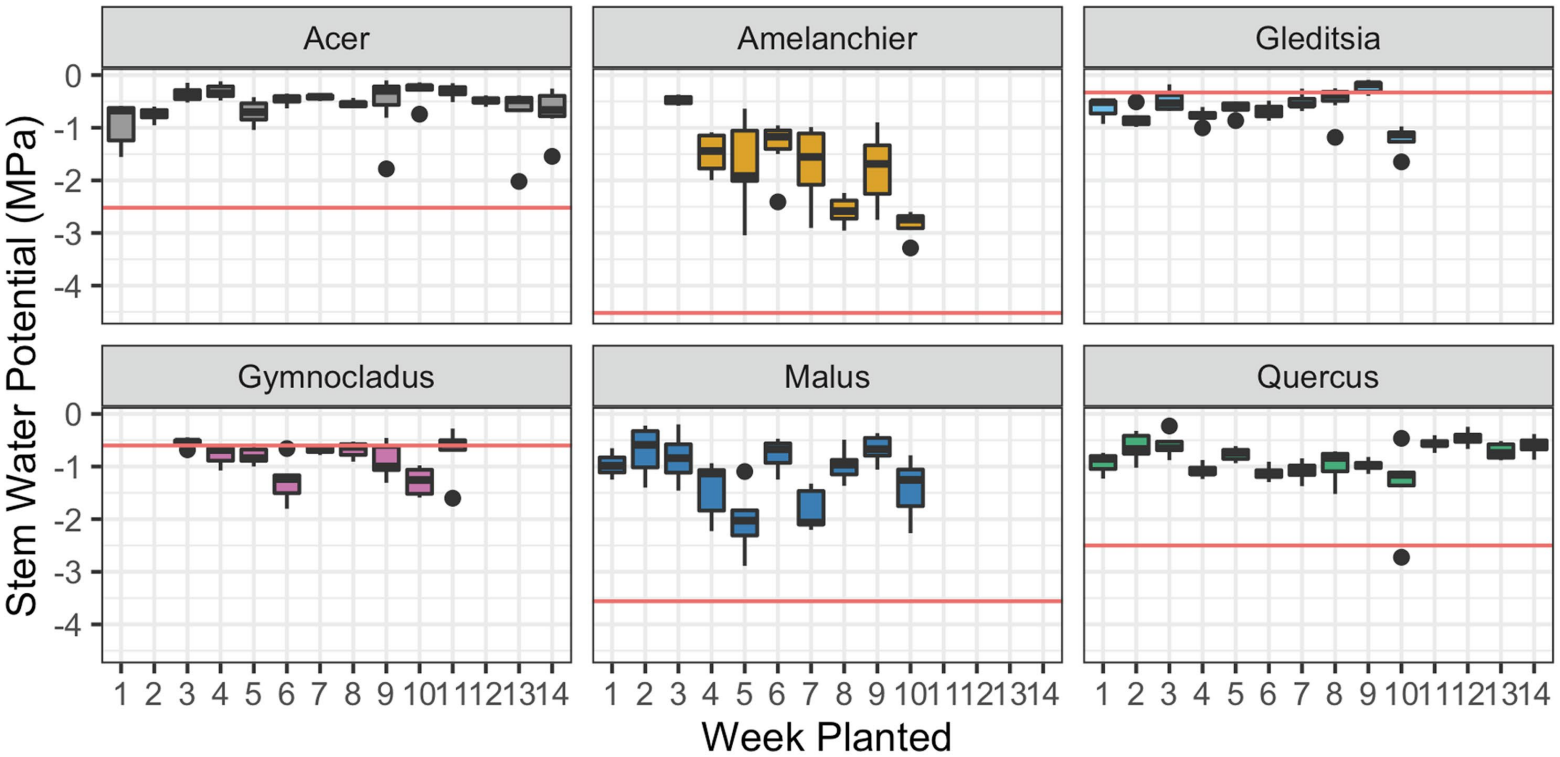
Stem water potential at the time of removal from cold storage and subsequent planting. Box plots show median, interquartile range, and minimum and maximum range for each week of planting. Solid red lines indicate stem *P*_50_ for each species, estimated by the stem vulnerability curves. There was evidence of a significant negative effect of planting date on stem water potential for *Amelanchier* (*p* < 0.01; details in text and Table 3). For all other cultivars, there was no evidence of a significant effect of planting date on stem water potential.

For one cultivar., *Amelanchier*, there was evidence of a significant negative effect of planting week on stem water potential ( β=−0.22 , *F*_1,51_ = 35.84, *p* < 0.01; Figure 3). *Amelanchier* was the cultivar we had the most logistical issues with procuring, so the effect is evaluated over only 8 weeks. *Amelanchier* also had the most negative stem *P*_50_, so despite a decline in stem water potential over time in cold storage, measured stem water potentials did not come close to exceeding stem *P*_50_ in the time frame we tested. For all other cultivars, there was no evidence of a significant effect of planting week on stem water potential (Table 3). Two cultivars—*Gleditsia* and *Gymnocladus*—had stem water potentials that exceeded stem *P*_50_ from the very first week of the experiment. For *Acer*, *Amelanchier*, *Malus*, and *Quercus*, median stem water potential did not exceed stem *P*_50_.

**Table 3:** Linear regression results for stem water potential, water content, total NSC, and incremental field growth for each cultivar.

	Stem ψ (MPa)				Stem WC (g/g)				Total NSC (mg/g DW)				Field Growth (cm)			
	β	*R* ^2^	*F*_df1, df2_	*p*	β	*R* ^2^	*F*_df1, df2_	*p*	β	*R* ^2^	*F*_df1, df2_	*p*	β	*R* ^2^	*F*_df1, df2_	*p*
*Acer*	0.01	0.01	1.14_1,96_	0.29	0.00	0.02	1.58_1,96_	0.21	7.73	0.17	3.96_1,19_	0.06	0.06	0.00	0.05_1,138_	0.82
*Amelanchier*	−0.22	0.41	35.85_1,51_	**<0.01**	0.00	0.00	0.20_1,51_	0.66	8.21	0.37	9.43_1,16_	**<0.01**	0.74	0.01	0.59_1,72_	0.44
*Gleditsia*	0.01	0.11	0.71_1,64_	0.40	0.01	0.03	1.91_1,65_	0.17	−13.25	0.05	0.83_1,16_	0.38	−0.36	0.00	0.25_1,93_	0.62
*Gymnocladus*	−0.03	0.04	2.21_1,57_	0.14	0.00	0.00	0.08_1,60_	0.77	−16.67	0.22	5.40_1,19_	**<0.05**	−0.06	0.00	0.08_1,88_	0.77
*Malus*	−0.03	0.02	1.05_1,68_	0.31	0.01	0.04	2.78_1,68_	0.10	6.31	0.04	0.75_1,19_	0.40	−1.67	0.10	10.91_1,98_	**<0.01**
*Quercus*	0.01	0.02	2.33_1,96_	0.13	0.00	0.01	0.81_1,96_	0.32	5.053	0.05	0.92_1,19_	0.35	−2.03	0.12	18.53_1,138_	**<0.01**

*N* varies by cultivar and metric, so degrees of freedom are given with the F-statistics. Results where p < 0.05 are highlighted in bold.

**Figure 4:**
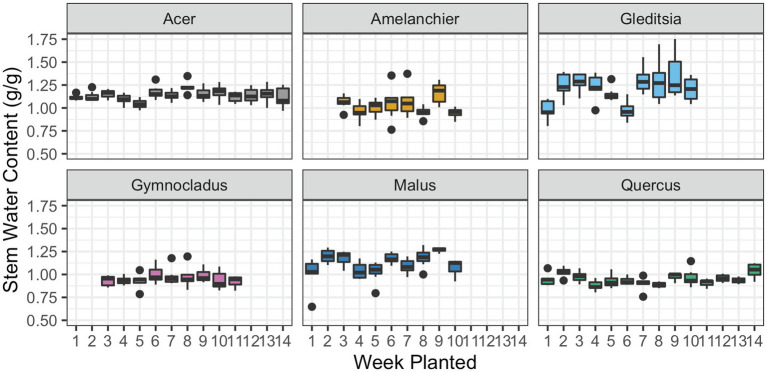
Stem water content at the time of removal from cold storage and subsequent planting. Box plots show median, interquartile range, and minimum and maximum range for each week of planting. One outlier for *Gleditsia* has been removed in this figure, but not the statistical analysis. There was no evidence of a significant effect of planting date on stem water content for any cultivar.

For all six cultivars, there was no evidence of a significant effect of the planting dates in the stem water content (Figure 4). The parameters of the linear regression model are given in Table 3. Stem water content is expressed in grams of water in fresh tissue to grams of dry plant tissue; with most stem water content values falling around or above 1 g/g, this indicates that the fresh tissue is over 50% water content, by weight.

**Figure 5:**
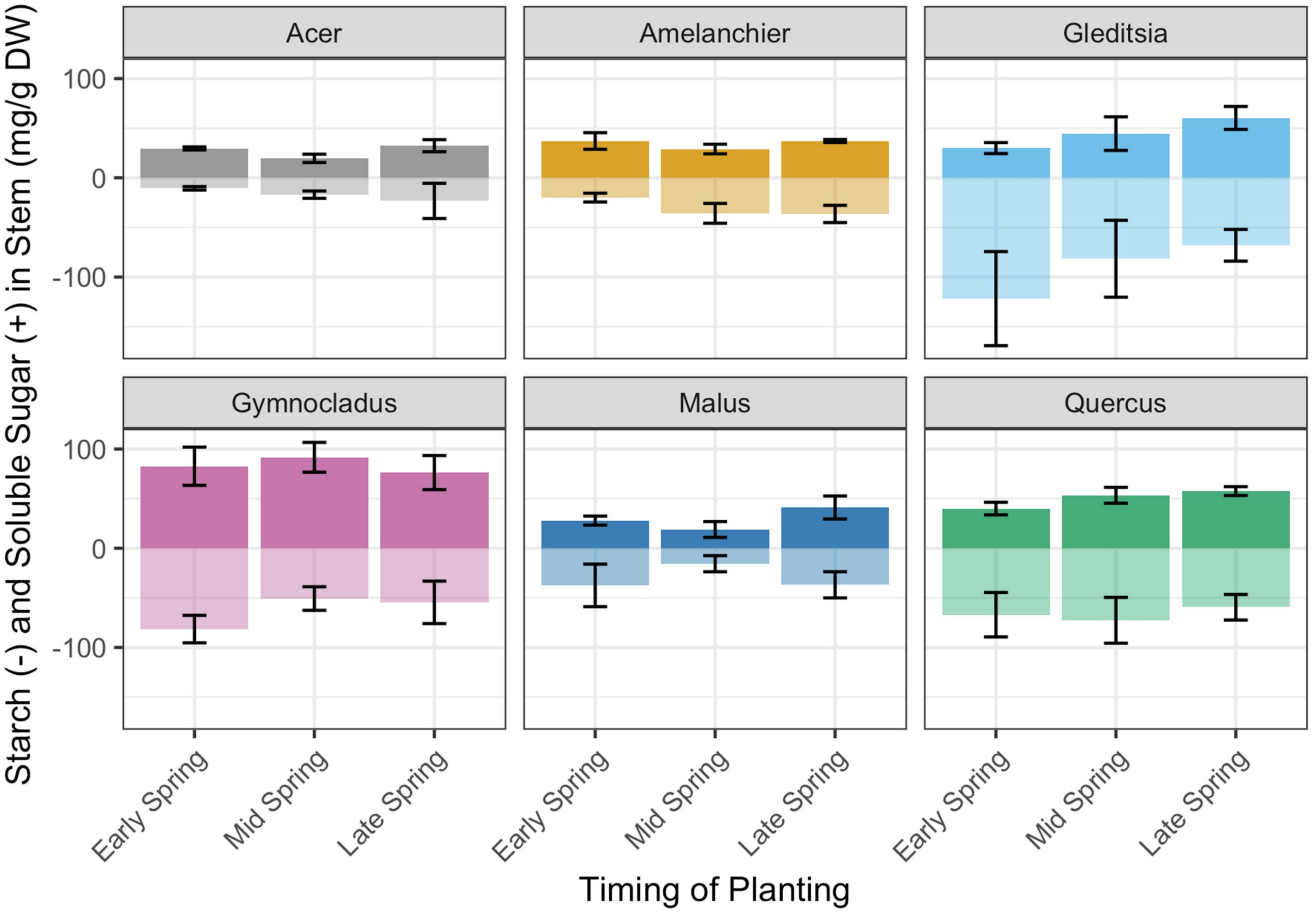
Total non-structural carbohydrate concentration and relative proportion of starch (transparent bars, below midline) and soluble sugars (solid bars, above midline). The specific dates for the timing of planting categories are in Table 1. There was evidence of a significant positive relationship of planting week on total NSC concentration for *Amelanchier* (*p* < 0.01; details in text and Table 1) and evidence of a significant negative relationship for *Gymnocladus* (*p* < 0.05; details in text and Table 1). Error bars indicate standard deviation for soluble sugar and starch concentration separately. Soluble sugars and starch concentrations were not tested separately in linear regression models.

Based on the expectation that total NSC would diminish over time in storage due to respiration, we evaluated the effect of the week of removal from cold storage and planting on total NSC concentration (mg/g DW). There was evidence of a significant positive effect of planting week on total NSC for *Amelanchier* ( β=8.21 , *F*_1,16_ = 9.43, *p* < 0.01). In contrast, for *Gymnocladus*, there was evidence of a significant negative effect of planting week on total NSC ( β=−16.68 , F_1,19_ = 5.40, *p* < 0.05). There was no evidence of a significant effect of planting week on total NSC for the other cultivars (Table 3). Among the cultivars, there was no clear pattern of a shift between starch concentration and soluble sugars concentration (Figure 5).

**Figure 6:**
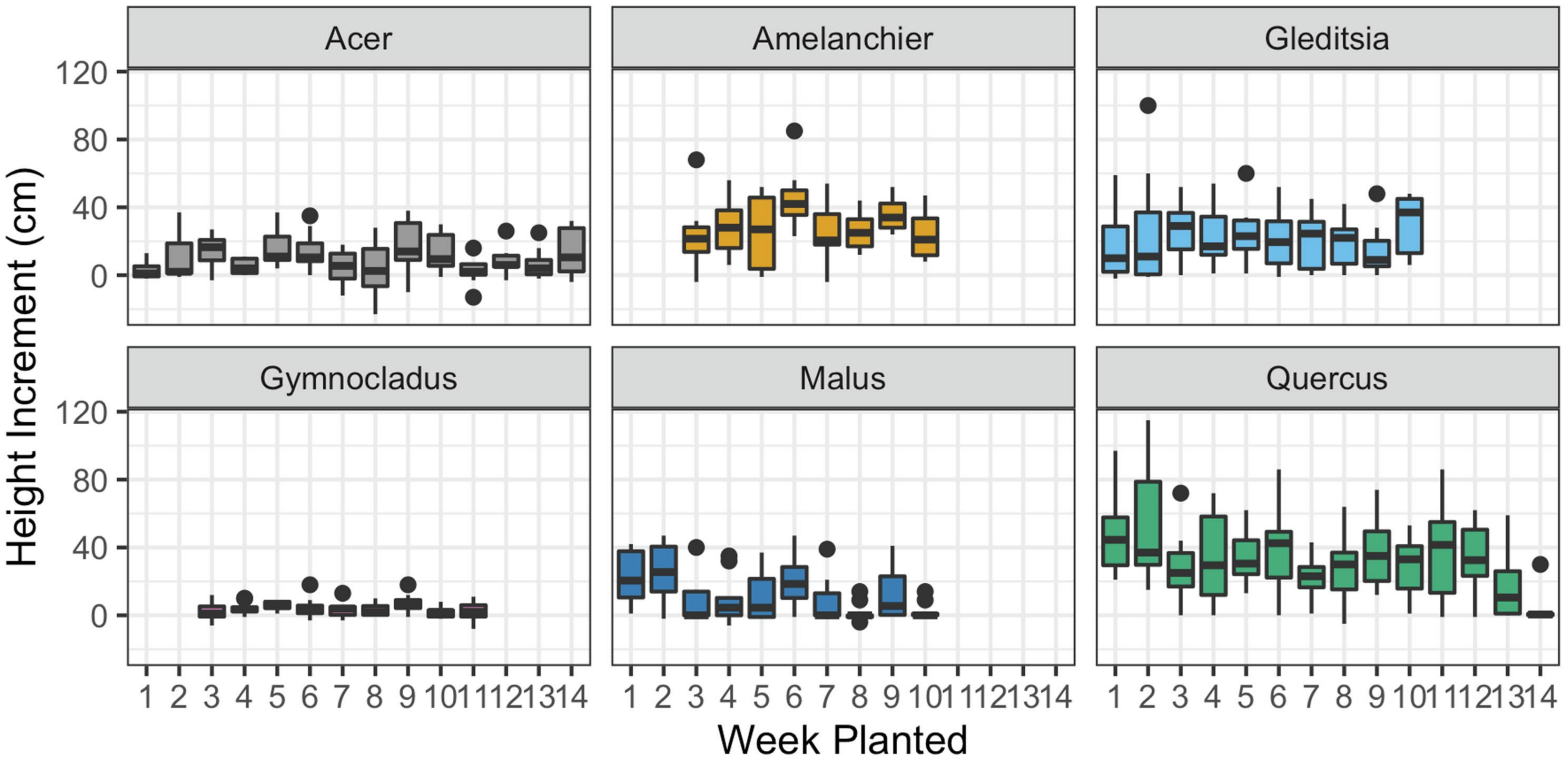
Incremental height growth by the time of plantings. Height was measured for each tree at planting and in late fall 2020 and the difference is given here. Box plots show median, interquartile range, and minimum and maximum range for each week of planting. Negative height increments suggest die-back of the terminal stem. There was evidence of a significant effect of planting date on height growth for *Malus* and *Quercus* (*p* < 0.01; details in text and Table 3).

In the case of four out of six of the cultivars, there was no significant effect of the planting date on the height growth in the season that followed planting (Figure 6; Table 3). There was evidence of a significant negative effect of planting date on height growth for *Malus* ( β=−1.67 , F_1,98_ = 10.91, *p* < 0.01) and *Quercus* ( β=−2.03 , *F*_1,138_ = 18.53, *p* < 0.01). The significant effect of planting date on height in these cultivars is driven by the absence of height growth in the latest-planted trees. Additionally, we observed frequent terminal stem die-back in planted *Malus*. However, there was not a higher incidence of mortality at the later planting dates. We observed that bud flush followed the timing of planting. None of the cultivars had broken bud in cold storage, but the *Acer* trees had bloomed in storage before the first planting date. At the end of the growing season, we observed that leaf senescence occurred across a gradient, beginning several days earlier in the early-planted trees than the late-planted trees.

## Discussion

Contrary to previous work, we did not observe the effects of extended cold storage on growth after planting for most of the cultivars we tracked in this experiment. For two cultivars, *Malus* and *Quercus*, there was a negative relationship between the timing of planting and incremental height growth, but this could not be tied to a change in water relations or carbon depletion. For all cultivars, survival was high when the trees were planted into an irrigated, weeded environment. The managed planting site probably helped tree performance: stressful environmental conditions at the planting site are more likely to expose detrimental impacts from the nursery production process (Grossnickle et al., 2020). However, the consistent height growth in the field helps to rule out other detrimental effects of extended storage on physiological traits that we did not measure within the experiment. The range of planting dates tested in this experiment extended from the operational standard, in early to mid-spring, to beyond what commercial growers would consider feasible, at the beginning of summer. In other research, extended time in cold storage and desiccation during storage have been tied to poor outplanting performance and changes in physiology for nursery-grown plants (Tung et al., 1986; Deans et al., 1990; Mena-Petite et al., 2001; Overton et al., 2013).

We expected that trees with a stem water potential that exceeded *P*_50_ or more in storage would have detrimental effects on growth and survival after planting. Hydraulic failure occurs when there is extensive xylem embolism, which reduces a stem’s capacity to conduct water. For angiosperm species, lethal water potentials correlated with 80–100% loss of xylem hydraulic conductance (PLC; Choat et al., 2018). Short of catastrophic hydraulic failure, chronically high PLC (e.g., P_60_) may also precede mortality (Sperry and Love, 2015). Therefore, *P*_50_ is a conservative threshold for stem water potentials beyond which we might expect to see detrimental effects to plant growth and survival. From the first measurements in mid-March, the stem water potential measurements in *Gleditsia* and *Gymnocladus* were below stem *P*_50_, as determined by the hydraulic vulnerability curves. However, these trees grew without issue after outplanting. The stem water potential measurements for the remaining four cultivars did not exceed stem *P*_50_ values. Though there was a negative relationship between *Amelanchier* and planting date, there were not detrimental effects observed in the growth of this cultivar.

For this experiment, the recovery of the hydraulic system in conjunction with spring growth is particularly important. The loss of stem hydraulic conductance during winter may not impact growth in all species, particularly ring-porous species. Spring recovery of hydraulic function can occur with growth of new xylem vessels and possibly by refilling of embolized conduits (Christensen-Dalsgaard and Tyree, 2014). Some trees can experience over 90% PLC by early spring, but regain stem conductance following leaf expansion and earlywood growth (Jaquish and Ewers, 2001; Christensen-Dalsgaard and Tyree, 2014). In ring-porous species, including the *Gymnocladus*, *Gleditsia*, and *Quercus* cultivars in this experiment, building a new tree ring in the spring allows for full recovery of hydraulic conductance (Urli et al., 2012). This is likely the reason that *Gymnocladus* and *Gleditsia* grew without issue after planting, despite experiencing stem water potentials in cold storage that exceeded *P*_50_.

However, the growth of new xylem is not the only strategy for hydraulic recovery. Diffuse-porous trees, represented by the *Malus*, *Acer*, and *Amelanchier* cultivars in this experiment, are more likely to rely on the development of positive xylem pressure for spring recovery of hydraulic conductivity (Hacke and Sauter, 1996; Niu et al., 2017). Trees held in cold storage maintained at a constant temperature may miss seasonal temperature changes that initiate physical processes that create xylem pressure (Schenk et al., 2021). Additionally, trees kept in storage before planting do not have access to soil water for the creation of positive root pressure, which can contribute to hydraulic recovery (Zhu et al., 2000; Jaquish and Ewers, 2001). Therefore, diffuse-porous trees might be particularly susceptible to damage if physiological thresholds are crossed in cold storage. In orchard settings, *Malus* cultivars at 70% PLC mid-winter had subsequent damage and die-back (Beikircher et al., 2016). High winter PLC in peach (*Prunus persica* Batsch) trees led to low rates of bud break as well as arrested growth of new shoots (Améglio et al., 2002). The *Malus* grown in our experiment experienced frequent terminal stem die-back. However, we did not observe a relationship between tip mortality and planting dates.

There is evidence to suspect that cultivated plants could be more vulnerable to hydraulic failure than wild-type species (Beikircher et al., 2013). Nursery practices, such as fertilization and irrigation, can change the xylem anatomy of plants, which impacts hydraulic function and vulnerability (Beikircher et al., 2019; Sloan et al., 2020). Grafting, rootstock choice, and selection for traits such as rapid growth will affect tree vigor, anatomy, and subsequently, hydraulic physiology and vulnerability to embolism (Beikircher et al., 2013). Plant growth is pushed in nursery production because larger plants are more valuable. Yet rapid growth, especially late-season, is less suberized and more at risk of desiccation than early-season growth and is more vulnerable to hydraulic failure (Beikircher and Mayr, 2013).

As was generally the case with stem water potential, we saw no trend in stem water content over the extended time in storage. Desiccation in cold storage represents a threat to nursery-grown trees, given the importance of stem water content to tree growth after planting. Through the winter, stem tissues can lose up to half of their water content, impacting the onset of water stress (Charrier et al., 2015). Many storage rooms in nursery production systems are essentially very large refrigerators and do not have humidity controls. A common practice is for growers to occasionally spray the plants with a hose or mulch with wetted shredded paper. Both methods are imprecise and can lead to desiccating conditions. Even though we did not see detrimental effects for the trees in this experiment, we still recommend nursery growers monitor the water content of roots and stems during the time in storage to track tree status. Water is needed in the stem before bud break for starch hydrolysis, cell growth, cell expansion, and the resumption of water flow through the stem if conductance has been lost (Améglio et al., 2002; Copini et al., 2019). Water is also necessary for mobilizing non-structural carbohydrates (NSC) reserves for survival and growth, another crucial component of winter survival (Tomasella et al., 2017). During the growing season, water stored in roots, branches, and leaves can buffer xylem tensions created by water stress and help preserve hydraulic function (Scholz et al., 2011; Cuneo et al., 2016; Knipfer et al., 2019); stored water during the dormant season may play a similar buffering role.

We did not see a consistent trend in total NSC concentrations among the cultivars for the time intervals at which we measured NSC. In the two cultivars where there was evidence of a significant effect of planting date on total NSC, yet the trends were contradictory, with a positive relationship between the metrics for *Amelanchier* and a negative relationship for *Gymnocladus*. While there are known methodological challenges to NSC measurements, the results reported here originate from one lab, allowing us to compare among them (Landhäusser et al., 2018). The expected pattern of NSC reserves through the winter starts with high levels at the end of the growing season, then a slow decline through the dormant season to meet respiratory demands, with a final rapid decline at leaf-out or earlywood formation (Dietze et al., 2014). Predictably, the starting NSC condition for plants in storage is an important factor for the carbon dynamics over time in storage (Christiaens et al., 2015). Extended time in cold storage means additional energy demands for maintenance respiration, which can impair outplanting performance (Cannell et al., 1990; Villar-Salvador et al., 2015). NSC was first measured in early spring, when the first trees were removed from cold storage and planted, so we did not capture the expected peak NSC concentration. Future studies on carbon dynamics in these cultivars would benefit from NSC measurements when trees are first put into storage.

In this experiment, the soluble sugars and starch concentrations were highly variable for all cultivars. The balance of starch and soluble sugars is also expected to shift over the dormant season: starch acts as a reservoir of energy for future use, while soluble sugars are used to perform immediate functions such as osmoregulation (Martínez-Vilalta et al., 2016). While reduced NSC levels may not be the ultimate cause of mortality, they can contribute to weakened trees that succumb to other stresses (Charrier et al., 2015). NSC reserves may have an osmotic role in stem pressure formation that leads to hydraulic recovery (Strati et al., 2003; Améglio et al., 2004; Yin et al., 2018; Zhang et al., 2018). Carbon depletion can also contribute to reduced water stress tolerance and slower resumption of growth (Jiang et al., 1995). Seedlings and saplings start with relatively small NSC reserves going into the dormant season, putting them at higher risk of mortality (Losso et al., 2018). On top of that, nursery trees face unique risks: pruning at the time of nursery lifting removes NSC sinks in the roots and branches (Charrier et al., 2015). If trees survive until planting, concerns about NSC reserves lessen: the leaves of deciduous trees can become autonomous from stored C reserves soon after bud break and do not rely on stored carbohydrates for long (Hoch et al., 2003).

Nursery-grown trees are important not only for the horticultural industry but also, in tree planting projects, one of our best solutions for addressing climate change (Bastin et al., 2019). Maintaining urban forests, wildland restoration, and reforestation are challenged by the environmental conditions associated with climate change, like extreme heatwaves, that make it more difficult for trees to survive after planting. Warm autumn temperatures delay dormancy and planting windows are more unpredictable (Beil et al., 2021; Fargione et al., 2021). Extreme weather puts even more pressure on nursery professionals to understand how plant physiology, including water relations and carbon dynamics, responds to each stage of the nursery production process (Sheridan and Nackley, 2021). An understanding of plant hydraulic physiology will also guide decisions about how to manage tree production and planting under warmer, drier future climate conditions (Losso et al., 2018). While we were not able to identify specific physiological thresholds or trends among the deciduous cultivars we tracked in this experiment, we present the first hydraulic response to cold storage data for these important genera and demonstrate how plant physiology can be integrated into nursery practices to guide plant production.

## Data Availability Statement

The raw data supporting the conclusions of this article will be made available by the authors, without undue reservation.

## Author Contributions

RS and LN: conceptualization, investigation, methodology, writing original draft, and writing-review and editing. RS: formal analysis and data curation. LN: funding acquisition. All authors contributed to the article and approved the submitted version.

## Funding

Funding for this project was provided in part by the Oregon Association of Nurseries and the Oregon Department of Agriculture nursery research program grant no. K11920.

## Conflict of Interest

The authors declare that the research was conducted in the absence of any commercial or financial relationships that could be construed as a potential conflict of interest.

## Publisher’s Note

All claims expressed in this article are solely those of the authors and do not necessarily represent those of their affiliated organizations, or those of the publisher, the editors and the reviewers. Any product that may be evaluated in this article, or claim that may be made by its manufacturer, is not guaranteed or endorsed by the publisher.
